# Exposure of tropical ecosystems to artificial light at night: Brazil as a case study

**DOI:** 10.1371/journal.pone.0171655

**Published:** 2017-02-08

**Authors:** Juliana Ribeirão de Freitas, Jon Bennie, Waldir Mantovani, Kevin J. Gaston

**Affiliations:** 1 IEE—Instituto de Energia e Ambiente, Universidade de São Paulo, São Paulo (SP), Brasil; 2 Centre for Geography, Environment and Society, College of Life and Environmental Sciences, University of Exeter, Penryn, Cornwall, United Kingdom; 3 Environment & Sustainability Institute, University of Exeter, Penryn, Cornwall, United Kingdom; University of Southern California, UNITED STATES

## Abstract

Artificial nighttime lighting from streetlights and other sources has a broad range of biological effects. Understanding the spatial and temporal levels and patterns of this lighting is a key step in determining the severity of adverse effects on different ecosystems, vegetation, and habitat types. Few such analyses have been conducted, particularly for regions with high biodiversity, including the tropics. We used an intercalibrated version of the Defense Meteorological Satellite Program’s Operational Linescan System (DMSP/OLS) images of stable nighttime lights to determine what proportion of original and current Brazilian vegetation types are experiencing measurable levels of artificial light and how this has changed in recent years. The percentage area affected by both detectable light and increases in brightness ranged between 0 and 35% for native vegetation types, and between 0 and 25% for current vegetation (i.e. including agriculture). The most heavily affected areas encompassed terrestrial coastal vegetation types (restingas and mangroves), Semideciduous Seasonal Forest, and Mixed Ombrophilous Forest. The existing small remnants of Lowland Deciduous and Semideciduous Seasonal Forests and of Campinarana had the lowest exposure levels to artificial light. Light pollution has not often been investigated in developing countries but our data show that it is an environmental concern.

## Introduction

The nighttime environment is undergoing a dramatic transformation across the Earth’s surface. The cycles of natural light (daily, lunar and seasonal) that have been major forms of environmental variation since the first emergence of life are being disrupted through the introduction of artificial lighting. A diversity of sources (including street lighting, advertising lighting, architectural lighting, security lighting, domestic lighting and vehicle lighting) are causing direct illumination as well as via skyglow, the scattering by atmospheric molecules or aerosols of artificial light at night that is emitted or reflected upwards [1–5].

Because natural cycles of light have previously provided rather consistent resources and sources of information for organisms, artificial nighttime lighting has a broad range of biological effects [5–7]. These span from gene to ecosystem levels [8,9]. They include effects on the physiology, behaviour, reproductive success and mortality of species (e.g. [10–13]), on their abundance and distribution [14], and in turn on community structures and functioning (e.g. [2,15]). Moreover, it seems likely that the impacts of artificial nighttime lighting interact with those of other pressures on biodiversity, including habitat loss, climate change, other forms of pollution, and invasive species [16].

Determining the severity of these biological impacts rests, in part, on understanding of the spatial and temporal levels and patterns of artificial nighttime lighting, and particularly how these interact with those of different ecosystem, vegetation and habitat types [16]. At a global scale, virtually all natural terrestrial ecosystem types experience some level of exposure to artificial nighttime lighting or skyglow, and those that have been most and least affected have been identified [4]. However, more detailed regional analyses have largely been wanting. A few evaluations exist of regional patterns of artificial nighttime lighting, but these have not tended to determine the interaction with ecosystem, vegetation, or habitat types (e.g. [15,17]). Of particular concern is that work on spatial patterns of artificial nighttime lighting has focussed predominantly on China, Europe and North America [1,3,17,18] with almost no attention to global biodiversity hotspots. In particular, the potential environmental impacts of artificial nighttime lighting in tropical regions have been surprisingly little considered.

Aside from the often much greater levels of biodiversity that could be influenced, it remains unknown whether artificial nightttime lighting has different impacts in tropical regions compared with temperate ones. Obvious differences between tropical and non-tropical regions that might be significant are the short and rather invariant tropical periods of twilight, relatively low proportions of crepuscular and cathemeral species in tropical regions [19], the greater specialisation in tropical regions of some interspecific interactions that are known to be susceptible to influences from artificial nighttime light (e.g. plant-pollinator; [20,21]), and the prevalence of terrestrial species using bioluminescence, which are known to be vulnerable to light pollution [22–24].

In this paper we determine the spatial and temporal patterns of artificial nighttime lighting across Brazil in relation to the distribution of vegetation types. Brazil makes a particularly valuable case study. As well as being the largest country in South America, it has the largest number of species of any country in the world for many major taxonomic groups [25], has high levels of species endemism, and two recognised global biodiversity hotspots [26]. Brazil also has the richest biodiversity of bioluminescent beetles in the world [27].

## Methods

### Light data

Following Bennie *et al*. [3], we used nighttime stable lights annual composite images, created with data from the Defense Meteorological Satellite Program’s Operational Linescan System (DMSP/OLS), downloaded from the National Oceanic and Atmospheric Administration archives (1992–2012, n = 21). These images capture upwardly reflected and directed nighttime light. The images are nominally at 1 km resolution, but are re-sampled from data at an equal angle of approximately 2.7 km resolution at the equator. These images cover spectral responses from 440 to 940 nm with the highest sensitivity in the 500 to 650 nm region. The spectral range encompasses the primary emissions from the most widely used sources for external lighting in Brazil: low pressure sodium (589 nm), high pressure sodium (from 540 nm to 630 nm) and mercury vapour (545 and 575 nm) [1,28].

Each pixel is represented by a digital number (DN) of between 0 and 63. Zero represents no detectable upward radiance, while brightly lit areas saturate at values of 63. Images were inter-calibrated and drift-corrected following the method of Bennie *et al*. [3]. An average calibrated image for both the first (1992–1996) and the last (2008–2012) five years was created and the difference was calculated. To assess the changes over the full period time, we considered pixels increasing or decreasing by more than a threshold of 3 DN units of difference between the averages of the first and last years. It was previously observed that over 94% of observed increases in DN of more than 3 units and over 93% of observed decreases of the same magnitude were consistently related to the directions of changes on the ground (e.g., expansion or contraction of urban and industrial areas) [3]. Following Gaston *et al*. [29] and Duffy *et al*. [30], we considered pixels as exposed to artificial light when they had values higher than 5.5 DN units. By using a threshold effectively twice the detection limit for change, we defined a conservative estimate of lit area and limited the extent to which dark sites may be classified as lit due to noise in the data set or calibration errors [29,30].

### Vegetation type data

We used the vegetation map produced by the Brazilian Institute for Geography and Statistics [31], which is recommended as a good basis to compare with data obtained from remote sensing images [32]. This map presents both original native vegetation and current vegetation and land cover. The former portrays the original vegetation classes in Brazil likely found at the time of Portuguese colonisation [31], and the latter describes the vegetation now present [31]. Original vegetation includes 24 wider classes while the current is more detailed, including 52 classes (Table 1). The shapefile was produced by IBGE—Brazilian Institute of Geography and Statistics and accessed through REDD-PAC website (http://www.redd-pac.org/new_page.php?contents=data.csv) in WFS (web feature service) format.

**Table 1:** Vegetation classification for Brazil according to IBGE (2012).

Forest	Ombrophilous Forest	Dense Ombrophilous Forest	Alluvial Dense Ombrophilous Forest
			Lowland Dense Ombrophilous Forest
			Sub-Montane Dense Ombrophilous Forest
			Montane Dense Ombrophilous Forest
		Open Ombrophilous Forest	Alluvial Open Ombrophilous Forest
			Lowland Open Ombrophilous Forest
			Sub-Montane Open Ombrophilous Forest
		Mixed Ombrophilous Forest	Montane Mixed Ombrophilous Forest
			High-montane Mixed Ombrophilous Forest
	Seasonal Forest	Semi-deciduous Seasonal Forest	Alluvial Semi deciduous Seasonal Forest
			Lowland Semi deciduous Seasonal Forest
			Sub-Montana Semi-deciduous Seasonal Forest
			Montane Semi-deciduous Seasonal Forest
		Deciduous Seasonal Forest	Lowland Deciduous Seasonal Forest
			Sub-Montane Deciduous Seasonal Forest
			Montane Deciduous Seasonal Forest
Non Forest		Campinarana	Forest Campinarana
			Woody Campinarana
			Shurbland Campinarana
			Grassland Campinarana
		Savanna	Forest Savanna
			Woody Savanna
			Parkland Savanna
			Grassland Savanna
		Steppe-savanna	Forest Steppe-savanna
			Woody Steppe-savanna
			Parkland Steppe-savanna
			Grassland Steppe-savanna
		Steppe	Woody Steppe
			Parkland Steppe
			Grassland Steppe
		Pioneer formation	Alluvial Areas
			Restinga
			Mangrove
Other	Ecotone	Campinarana/Ombrophilous Forest	Campinarana/Ombrophilous Forest
		Steppe/seasonal Forest	Steppe/seasonal Forest
		Seasonal Forest /Primary Formations	Seasonal Forest /Primary Formations
		Dense Ombrophilous Forest/Mixed Ombrophilous Forest	Dense Ombrophilous Forest/Mixed Ombrophilous Forest
		Ombrophilous Forest/Seasonal Forest	Ombrophilous Forest/Seasonal Forest
		Steppe savanna /Seasonal Forest	Steppe savanna /Seasonal Forest
		Savanna/Seasonal Forest	Savanna/Seasonal Forest
		Savanna/Ombrophilous Forest	Savanna/Ombrophilous Forest
		Savanna/Primary Formations	Savanna/Primary Formations
		Savanna/Steppe-savanna	Savanna/Steppe-savanna
		Savanna/Steppe-savanna/Seasonal Forest	Savanna/Steppe-savanna/Seasonal Forest
	Relict Vegetation	Relict Vegetation	High-montane Relict Vegetation
			Montane Relict Vegetation
	Water	Water	Coastal Water Mass
			Continental Water Mass
	Rocky Outcrops	Rocky Outcrops	Rocky Outcrops
——			Agriculture
			Secondary Vegetation

The third column corresponds to original vegetation and the fourth column to current vegetation.

The IBGE map divides vegetation into two broad classes: forests and non-forests [33]. Forests are divided into Ombrophilous Forest and Seasonal Forest. The former is further divided into three physiognomies (Dense, Open and Mixed) and the last into two (Deciduous and Semi-deciduous). All of these can be classified by up to five formations: Alluvial, Lowland, Sub montane, Montane and High-montane (Table 1). Non-forests are divided into four formations: Campinarana, Savanna, Steppe-savanna, and Steppe, which in turn can be divided into up to four formations: Forest, Woody, Shrubland, and Grassland. The map also classifies pioneer formations—that encompass vegetation influenced by rivers (Alluvial Areas), by the sea (Restingas), and by both (Mangroves)—Ecotones, Relict Vegetation and Water. When considering the current vegetation, it also includes Agriculture and Secondary Vegetation classes (Table 1).

### Processing

To define the proportional area of each vegetation type that has been exposed to artificial nighttime light, we overlaid both original and current vegetation shapefiles on the DMSP data for the most recent five years (2008–2012). We extracted both the number of lit pixels and the total number of pixels inside each vegetation type and divided the first by the second. To assess changes, we overlaid the two vegetation shapefiles on the difference between the first (1992–1996) and the last (2008–2012) five years of DMSP data. We extracted the number of increasing pixels, decreasing pixels and the total number of pixels inside each vegetation type. We divided the number of increasing and decreasing pixels by the total in each vegetation type, achieving the proportional area where artificial light has been increasing and decreasing respectively.

## Results

**Fig 1:**
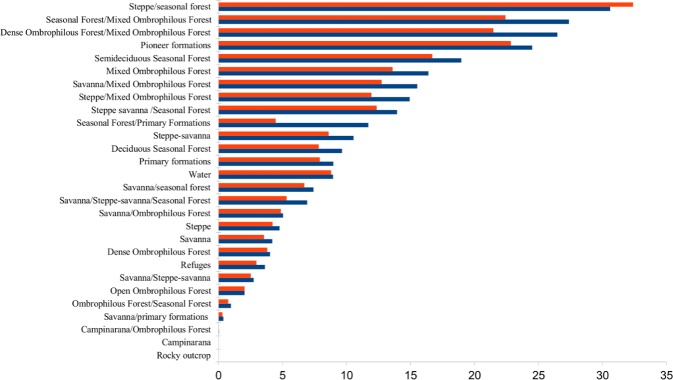
Percentage of area of original vegetation types affected by artificial light. Horizontal bars show the percentage of total land surface area occupied by each original vegetation type that had more than 5.5 Digital Number (DN) units in 2008–2012 (red) or an increase of more than 3 DN units between 1992–2012 and 2008–2012 (blue).

**Fig 2:**
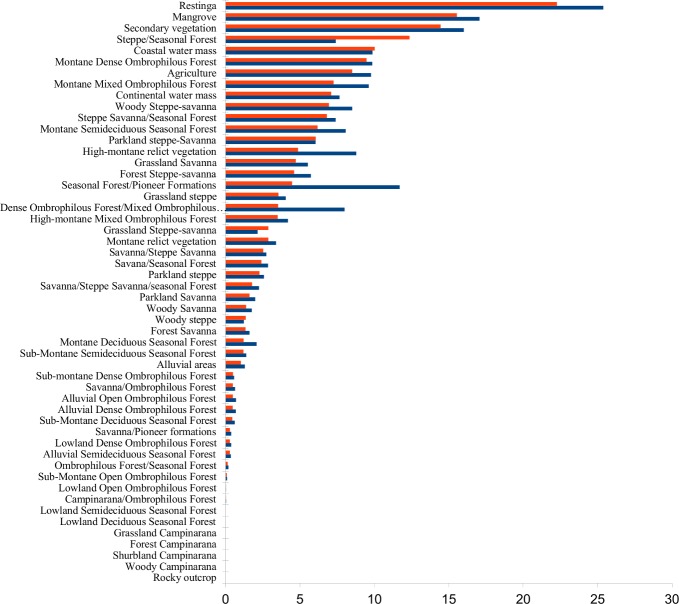
Percentage of area of current vegetation types affected by artificial light. Horizontal bars show the percentage of total land surface area occupied by each current vegetation type that had more than 5.5 Digital Number (DN) units in 2008–2012 (red) or an increase of more than 3 DN units between 1992–2012 and 2008–2012 (blue).

Overall, the percentage of area of each vegetation type affected by increases in artificial light was higher than the percentages affected by ‘detectable’ light (Figs 1 and 2). Less than 0.00001% of the areas of vegetation types experienced decreases in brightness so we considered only the increases in the results.

**Fig 3:**
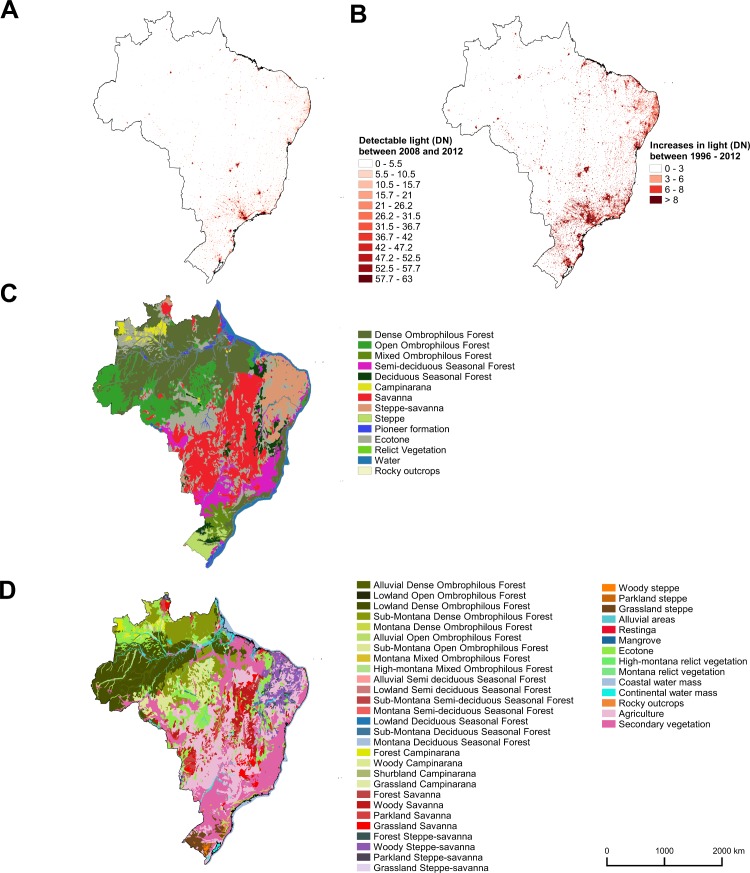
Spatial distribution of artificial light and vegetation types in Brazil. Distribution of: (A) pixels with detectable light (DN > 5.5) in the most recent five years (2008–2012); (B) pixels with increases in brightness (differences higher than 3 DN) between the first (1992–1998) and the last (2008–2012) five years; (C) original vegetation types; and (D) current vegetation types. The figure was created using QGIS 2.12.3. Nighttime light images were created with data from the Defense Meteorological Satellite Program’s Operational Linescan System (DMSP/OLS), freely available at the website of National Oceanic and Atmospheric Administration/National Geophysical Data Center (NOAA/NGDC) Earth Observation Group (http://ngdc.noaa.gov/eog/). The shapefile of Brazilian vegetation types was produced by IBGE (Brazilian Institute of Geography and Statistics) and is freely available at REDD-PAC website (http://www.redd-pac.org/new_page.php?contents=data.csv) in WFS (web feature service) format.

Spatial distribution of detectable light and increases in brightness followed similar patterns. The most affected areas were strongly concentrated along the coast, in the east, particularly in the southeast, while less affected areas were located in the west and in the central region (Fig 3A and 3B).

### Pre-colonization native vegetation

The area of original vegetation types affected by both detectable light and increases in brightness ranged between 0% and approximately 35%. Types affected by detectable light in more than 10% of their areas include pioneer formations (which encompass Mangroves, Restingas, and Alluvial Areas—Table 1), Semideciduous Seasonal Forest, Mixed Ombrophilous Forest, and six ecotones containing these ones and also Savanna, Steppe-savanna, Dense Ombrophilous Forest, and Steppe (Fig 1).

Less than 1% of the areas of three original vegetation types were affected by both detectable light and increases in exposure: Campinarana/Ombrophilous Forest, Savanna/ Pioneer Formations, and Ombrophilous Forest/Seasonal Forest (Fig 1). Two out of 24 original vegetation types had levels of detectable artificial light at night below the threshold: Rocky Outcrops and Campinarana (Fig 1). The less affected original vegetation types were concentrated in the west and in the central area while the most affected were in the southeast and northeast (Fig 3A and 3C).

### Current vegetation

The area of current vegetation types affected by detectable light ranged between less than 1% and approximately 25%. Restingas, Mangroves, Secondary Vegetation, and Steppe/Seasonal Forest had more than 10% of their areas affected by detectable light (Fig 2). The first three were also the most affected by changes in brightness as well as Seasonal Forest/ Pioneer Formations (Fig 2).

Vegetation types with less than 1% of their areas affected by both detectable light and increases in exposure were the three formations of Open and Dense Ombrophilous Forest (Alluvial, Lowland and Sub-montane—Table 1), Alluvial Semideciduous Seasonal Forest, Sub-Montane Deciduous Seasonal Forest and four ecotones involving Savanna, Ombrophilous Forest, Pioneer Formations (mainly Mangroves and Restingas), Campinarana and Seasonal Forest (Fig 2).

100% of the areas of seven of the 52 current vegetation types had levels of detectable artificial light lower than the threshold: Rocky Outcrops, the four formations of Campinarana (i.e. Woody, Shrubland, Forest and Grassland—Table 1), Lowland Deciduous Seasonal Forest and Lowland Semideciduous Seasonal Forest (Fig 2).

The most affected current vegetation types were strongly concentrated along the coast, in the east. The less affected ones occurred in the west (where Amazonia rainforest is located) and in the central area (Fig 3B and 3D).

## Discussion

In this paper we provide the first assessment of the broad level of exposure of tropical and subtropical ecosystems to artificial light at night at a regional extent. Because the percentage of areas of the different vegetation types affected by increases in brightness was higher than those affected by detectable light in most of the cases (Figs 1, 2, 3A and 3B), it seems inevitable that the extent of artificial lighting will continue to increase.

The highest aggregations of artificial lights in Brazil are in the coastal regions (Fig 3A and 3B) from where occupancy of Brazilian territory by Europeans started and where the larger urban agglomerations are now located [34]. The three most widely lit vegetation types when considering original vegetation are ecotones and all of them involve Seasonal Forest or Mixed Ombrophilous Forest (Fig 1). Semideciduous Seasonal Forest and Mixed Ombrophilous Forest themselves are also widely lit by detectable light (16.7% and 13.6% respectively—Fig 1). These levels of coverage by artificial lighting are lower for current vegetation of the same types (6.18% for Montane Semideciduous Forest, 1.2% for Sub-montane Semideciduous Forest, 7.25% for Montane Mixed Ombrophilous Forest, and 3.5% for High-montane Mixed Ombrophilous Forest) because they have been highly converted and the current remnants are small [35]. Of the current vegetation types, Restingas, Mangroves and Coastal water mass are among the five with the greatest percentage coverage by artificial nighttime lighting (Fig 2).

Imagery of emissions of upward radiance are the best available data to assess both the presence and trends in artificial light at a regional scale (other artificial nighttime lighting data sets do not yet capture trends). However, as pointed by Bennie *et al*. [4], trends established using these data must be interpreted with caution because the relationships between the images captured by the satellites and biologically relevant levels of light experienced by species are not straightforward. First, the spectral response of the OLS instrument covers the ranges of the most commonly used sources for external light, which differs from the action spectra of biological processes depending on the species. Second, because DMSP/OLS images are approximately at 2.7 km resolution, the correspondence between the illuminated areas in the images and the areas at the ground surface where biologically significant levels of lights are present is not precise. And finally, upwards radiance measures do not encompass horizontal emissions or skyglow–although it is important to observe that empirical data on temporal trends in the spatial occurrence of skyglow at continental scales are not presently available, and modelled surface data have large uncertainties [36,37].

Whilst an impressively wide array of ecological impacts of artificial nighttime lighting have been documented (see Introduction), the most important effects on given vegetation types and their associated communities remain unknown. Nonetheless, Semideciduous Seasonal Forest may potentially be differentially impacted because the trees lose from 20% to 50% of their leaves during the unfavourable season (i.e. dry and cold season in tropical and subtropical zones respectively [30]) and street lighting has previously been shown in other contexts to affect leaf fall timing as well as the speed of leaf growth [38,39]. Mixed Ombrophilous Forest, also known as araucaria forest due to the dominance of Brazilian pine (*Araucaria angustifolia*) [33], has a notably high richness and diversity of dung beetles [40]. It is known that dung beetles exploit moonlight, the celestial polarization pattern and the starry sky for orientation [41–44]. Given the important role of dung beetles in decomposition and nutrient cycling in tropical ecosystems, it seems likely that the high levels of artificial light and increase in brightness found in Ombrophilous Mixed Forest will affect its functioning.

Both Restinga and Mangrove are heavily overlapped by artificial light. Restinga is the terrestrial pioneer vegetation that occurs on sandy shore environments, especially on dunes, and is directly influenced by the sea [33]. Restinga harbours a high diversity of bats [45–47], which are known to be important for the maintenance of forests and to be disturbed by artificial light [48–50]. Around the world, mangroves are threatened by deforestation, illegal shrimp culture, expansion of urban areas, tourism, fishing and pollution [51]. Nine percent of the global area of natural or semi natural mangroves has seen an increase in exposure to artificial light [4]. In Brazil this percentage is 17% in the same period (Fig 2), with more than 15% of the mangrove area experiencing detectable light (Fig 2). Given that Brazil accounts for approximately 50% of mangroves in South America and 7% of the world’s mangroves [51], light pollution in these areas should be of particular concern. Both Restinga and Mangrove are coastal ecosystems and the coastal water mass itself is also highly affected by light (Fig 2). Five out of seven extant species of marine turtles in the world nest on the Brazilian coast (*Chelonia mydas*, *Caretta caretta*, *Dermochelys coriacea*, *Eretmochelys imbricata*, and *Lepidochelys olivacea*)—all of them are listed as threatened on the IUCN Red List (http://www.iucnredlist.org/search). Artificial lighting disrupts sea turtle hatchling orientation from the nest to the sea [52]. To protect Brazilian coastal ecosystems, the law forbids illumination within 50 m of the beach strip between Rio de Janeiro and Rio Grande do Norte States—which corresponds to approximately 2 500 km out of the 7 367 km of Brazilian coast [53]. Due to the scarcity of studies on the consequence of light pollution in these ecosystems, it is not possible to assess if the law is effective.

In most developing countries artificial nighttime lighting is relatively recent and concentrated in dense populated urban areas [37]. In contrast, in highly industrialised countries it is much more widespread [1,4], and often considered thus to be a much greater concern. However, our results here highlight that lighting is extensive in some developing countries, including ones with exceptionally high levels of biodiversity. These results also suggest that it is still possible to find vegetation types with natural sky background brightness. Countries in which this is the case have the opportunity to base policies, regulations, and guidelines on minimising rather than mitigating the ecological impacts of artificial nighttime lighting.
